# Diagnostic challenges and burden of idiopathic hypersomnia: a systematic literature review

**DOI:** 10.1093/sleepadvances/zpae059

**Published:** 2024-08-16

**Authors:** Talia Boulanger, Pascale Pigeon, Stephen Crawford

**Affiliations:** Talia Boulanger, LLC, Westford, MA, USA; Talia Boulanger, LLC, Westford, MA, USA; Takeda Development Center Americas, Cambridge, MA, USA

**Keywords:** idiopathic hypersomnia, central disorder of hypersomnolence, burden of illness, systematic literature review, diagnostic criteria, comorbidities, clinical burden, humanistic burden, economic burden

## Abstract

Idiopathic hypersomnia (IH) is a rare neurological sleep disorder, characterized by excessive daytime sleepiness despite normal sleep duration, that can significantly impact patient’s lives. The burden of IH goes beyond excessive daytime sleepiness, pervading all aspects of everyday life. Characteristic and burdensome symptoms of IH include sleep inertia/drunkenness, long sleep duration, and daytime cognitive dysfunction. This systematic review assessed current knowledge regarding IH diagnostic challenges and burden of illness. Literature searches for original epidemiological, clinical, humanistic, or economic research relevant to IH published between 2012 and 2022 in MEDLINE, Embase, Cochrane, gray literature (diagnostic criteria and treatment guidelines), conferences (2019–2022), and clinical trial databases yielded 97 articles. Findings indicate that IH remains a poorly defined diagnosis of exclusion that is difficult to distinguish from narcolepsy type 2 because of symptom overlap and inadequacies of objective testing. Consequently, individuals with IH endure diagnostic delays of up to 9 years. The economic burden of IH has not been characterized to any appreciable extent. Pharmacological treatment options can improve symptoms and functional status, but rarely restores normal levels of functioning. These findings highlight the need to reclassify central disorders of hypersomnolence. Further collaboration is now required between research groups to identify and validate objective markers to help redefine diagnostic criteria for IH. This would move IH into a position that could benefit from future targeted therapeutic interventions. The study was funded by Takeda Development Center Americas, Inc.

## Graphical Abstract

**Graphical Abstract:**
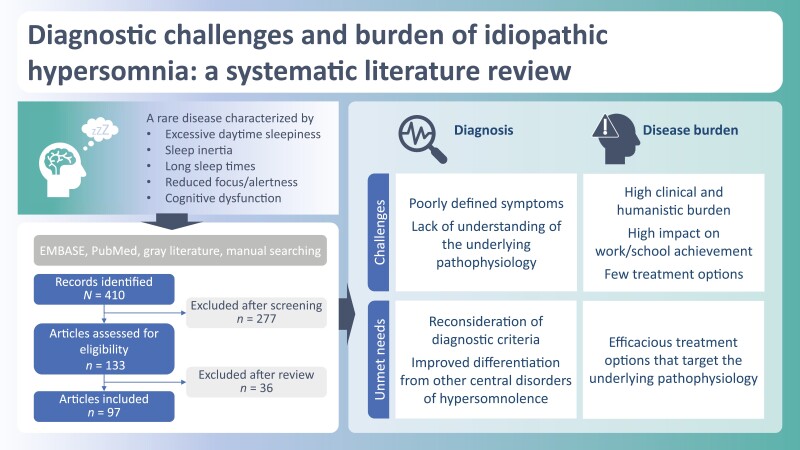

**Statement of Significance**

Idiopathic hypersomnia (IH) is widely considered to be a rare disease characterized by excessive daytime sleepiness, including difficulty waking up and reduced focus/alertness and mental fatigue, with important consequences for those affected. This review investigates the collective burden of IH as current information is lacking due to a previous focus on specific individual aspects of burden. The findings provide a better understanding of the challenges associated with diagnosing IH as well as the burden. Future work to identify and validate objective markers to inform re-classification of IH would help reduce delays in reaching a diagnosis and increase the potential for treatment through targeted intervention.

Idiopathic hypersomnia (IH) is widely considered to be a rare, chronic, neurological disorder of excessive daytime sleepiness (EDS) without cataplexy, in the absence of another identified cause [1–4]. The cardinal EDS symptom is defined as “an uncontrollable need to sleep or daytime sleepiness that persists for at least 3 months even with adequate or prolonged nighttime sleep” [1]. The length of nighttime sleep can exceed at least 9 hours (often more than 10 hours) and naps more than 1 hour, with both nighttime sleep and naps nonrestorative [1, 5]. Other characteristics of IH are sleep inertia/drunkenness (difficulty waking up) and impaired daytime function (reduced focus, reduced alertness, and fatigue) [5, 6]. IH can have a highly variable, lifelong clinical course after onset, and results in significant patient impact [1–4]. Onset usually occurs during adolescence or early adulthood (but can begin in infancy or as late as the mid-30s) [7–16], triggered by a wide range of factors including acute conditions and external life stressors [17]. Most prevalence estimates for IH range from 2 to 10.3 per 100 000 people, depending on the criteria and definitions used to determine cases [14, 18, 19].

IH is one of a group of disorders presenting with EDS, termed central disorders of hypersomnolence (CDHs), which also includes narcolepsy types 1 and 2 (NT1 and NT2), Kleine-Levin syndrome, insufficient sleep syndrome, and hypersomnia associated with a medical disorder, psychiatric disorder, or medication/substance [1, 4, 20–22]. Of the CDHs, NT1 is the most studied and best defined [5, 23]. NT2 and IH lack the specific symptoms of NT1, while their nonspecific symptoms (EDS related, sleep paralysis, and hallucinations) overlap [1, 4]. Although the majority of IH diagnoses are made by sleep medicine specialists, most often sleep neurologists, differential diagnosis of IH, in particular from NT2, can be challenging [1, 24]. IH presentation so closely resembles that of NT2 (presence of EDS, no cataplexy, no deficiency in cerebrospinal fluid orexin) that some authors have suggested combining IH and NT2 into “narcolepsy spectrum disorder” [25–30]. However, the etiology and pathophysiology of both IH and NT2 remain enigmatic, and it is unclear whether these disorders are related.

IH is associated with significant burden and can impact every aspect of patients’ lives [6]. Previous systematic reviews of the published literature have separately focused on single aspects of the burden of IH, but its collective burden has not been investigated adequately [14, 31–35]. This may stem from the difficulty in defining IH. We systematically reviewed the recent literature on IH to gain a better understanding of the challenges in diagnosing IH and the associated IH burden.

## Methods

The systematic literature review was conducted and reported in line with criteria stipulated by the Preferred Reporting Items for Systematic Review and Meta-Analyses Protocols (PRISMA-P) recommendations [36]. In addition to the yield obtained by the systematic literature search, narrative reviews were evaluated for context.

### Search strategy

Search algorithms included keywords and subject terms for IH and its variants (e.g. idiopathic hypersomnolence, primary hypersomnia) (Supplementary Tables S1 and S2). The databases searched were MEDLINE (via PubMed), Embase, citable gray literature (specifically, diagnostic criteria and treatment guidelines in IH sourced from government agencies or disease organizations), ClinicalTrials.gov, the World Health Organization’s International Clinical Trials Registry Platform, the Cochrane Library, and the World Sleep Congress 2019 abstracts (not indexed in Embase). The specific search terms and Boolean strategy for the PubMed and Embase searches are provided in Supplementary Tables S1 and S2. The searches were conducted in February 2022.

Bibliographies of full-text systematic reviews and meta-analyses identified by the search were reviewed manually to identify any additional articles meeting inclusion criteria.

### Eligibility criteria

Eligible articles had abstracts linked to full text or posters reporting original epidemiological, clinical, humanistic, or economic research on IH published in the last 10 years (or the last two conferences for conference abstracts). Primary studies, meta-analyses, systematic literature reviews, and guidelines were included; narrative reviews, editorials, and case reports were excluded (Supplementary Table S3). All articles pertained to humans, with animal or in vitro studies excluded.

### Screening and data collection

Titles and abstracts captured from the searches were compiled to create a single list of references for initial screening against the eligibility criteria and search topic. All articles were required to report on burden of illness of IH, including disease presentation, clinical burden, treatment, humanistic burden, and economic burden. Papers were excluded if they were not relevant to IH or did not report IH information separately from other conditions. All articles deemed relevant after abstract screening underwent full-text screening, which was conducted by two independent reviewers, with discordance resolved by discussion or a separate reviewer. Data were extracted from each eligible publication using a standardized Microsoft Excel form developed for the systematic review. Extracted data included those related to study design, sample (size, type, and source), and type of information on IH.

### Quality assessment

Oxford Levels of Evidence, from the Centre for Evidence-Based Medicine, were used to score study quality [37].

## Results

### Search results

**Figure 1.**
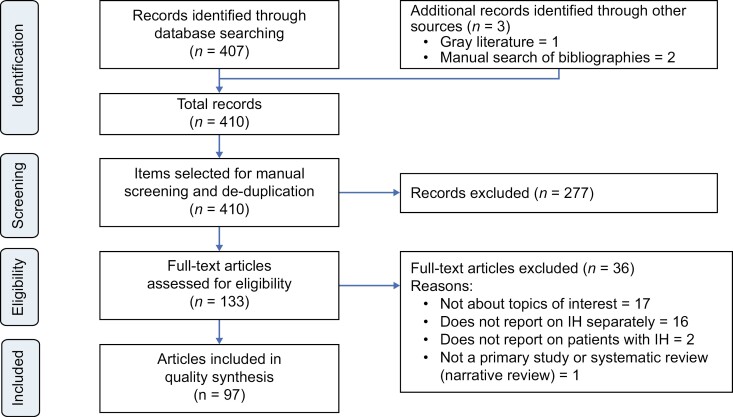
Literature identification and study selection process for publications reporting on idiopathic hypersomnia (IH) (PRISMA flowchart) [36].

The literature searches identified 410 articles (Figure 1). During title and abstract screening, 277 articles were rejected according to study exclusion criteria. A total of 133 articles were retrieved in full text, of which 36 were excluded, primarily due to lack of relevance to IH and conflation between patients with IH and other CDHs. Thus, 97 articles met the study eligibility criteria (aligning to Oxford Levels of Evidence grades 1–3) and were included in the review (Supplementary Table S4). Of these, three were guidelines, six were systematic literature reviews/meta-analyses, and 88 were original research studies (Supplementary Table S4). The 88 primary studies comprised six randomized controlled trials, one controlled clinical trial, 26 prospective cohort studies, seven prospective/cross-sectional studies, eight cross-sectional studies, three retrospective and prospective cohort studies, 34 retrospective studies, and three case-control studies (Supplementary Table S4). Of the 88 primary studies, 7 examined pediatric populations, while 22 involved either a small proportion of patients under 18 years of age or reported standard deviations in the age ranges suggestive of a few patients being minors. One systematic literature review focused on children and adolescents with either narcolepsy or IH. No studies reporting societal or direct costs, or resource utilization, specific to IH were published in the last 10 years; the only economic publications identified were related to productivity and employment.

### Diagnostic challenges

#### Current diagnostic criteria

Among the published articles included in this systematic literature review, the most frequently cited diagnostic criteria for defining and classifying IH are from the International Classification of Sleep Disorders (ICSD), Third Edition (ICSD-3), published in 2014 [1], with a more recent text revision (ICSD-3-TR) published in 2023 [38]. Since inception of the ICSD in 1990, there have been significant advances in the understanding of sleep disorders that have resulted in changes in their classification. An ICSD-3 diagnosis of IH requires the presence of EDS not better explained by any other cause (e.g. other sleep disorder or medication side effects), a Multiple Sleep Latency Test (MSLT) ≤8 min, and zero or one sleep-onset rapid eye movement periods (SOREMPs) (Table 1). IH can also be diagnosed if an otherwise well-rested person sleeps for ≥11 hours per 24-hours period, as estimated by either polysomnography (PSG) or wrist actigraphy and a sleep diary averaged over 1 week [1, 29, 39]. Diagnostic criteria defining IH from the Diagnostic and Statistical Manual of Mental Disorders, Fifth Edition (DSM-5), requires the exclusion of a mood disorder before IH can be diagnosed [40]. DSM-5 requires EDS at least three times per week (rather than daily per ICSD-3) and distinguishes between IH variants with and without long nocturnal sleep using a cutoff of 9 hours [1, 29, 39, 40]. Of note, ICSD Second Edition criteria made a distinction between IH subtypes with and without long sleep duration [41], which was omitted in ICSD-3 because of insufficient supporting data [1].

**Table 1.** Summary of the International Classification of Sleep Disorders (Third Edition-Text Revision) Criteria for Diagnosis of NT1, NT2, and IH [1, 38]

NT1	NT2	IH
Irrepressible need to sleep, or lapses into sleep during the daytime, daily for ≥3 months		
Cataplexy and either (a) mean sleep latency≤8 min and ≥2 SOREMPs on MSLT or (b) a SOREMP ≤15 min of sleep onset on the preceding nocturnal PSG, and/or CSF orexin concentration below normal (i.e., <110 pg/mL or <1/3 control values)	Mean sleep latency ≤8 min and ≥2 SOREMPs on MSLT (or REM ≤15 min of sleep onset on nocturnal PSG may replace 1 SOREMP)	<2 SOREMPs on MSLT, or 0 SOREMPs if REM ≤15 min of sleep onset on nocturnal PSG 1. Mean sleep latency ≤8 min on MSLT, and/or 2. ≥11 hours sleep time in 24 hours on either: a.PSG or b. wrist actigraphy plus sleep log, averaged over ≥7 days 3. PSG and MSLT findings are not consistent with a diagnosis of narcolepsy type 1 or 2
	No cataplexy Hypersomnolence and/or MSLT findings are not better explained by other causes, including insufficient sleep syndrome CSF orexin concentration normal if measured^*^	

CSF, cerebrospinal fluid; IH, idiopathic hypersomnia; MSLT, multiple sleep latency test; NT1, narcolepsy type 1; NT2, narcolepsy type 2; PSG, polysomnography; REM, rapid eye movement; SOREMP, sleep-onset rapid eye movement period.

^*^Normal orexin concentration ≥110 pg/mL or ≥1/3 control values.

#### Challenges to current diagnostic criteria

ICSD-3 has been critiqued within the published literature regarding its ill-defined classification of CDHs, which have overlapping signs and symptoms, especially between the narcolepsies and IH (Table 2) [23, 29, 53]. The ICSD-3 definition of IH has been called “a condition where excessive sleepiness has no obvious explanation, unreliable objective measures, and no convincing biological markers” [17]. The diagnostic criteria also do not provide levels or grades of certainty for evidence supporting CDH diagnoses, adequately grade the severity of sleep-related symptoms, or state how to reliably measure and assess sleep deprivation and circadian rhythm disturbances when ruling them out in the process of diagnosing IH [53].

**Table 2.** Symptoms typically associated with the narcolepsies (NT1 and NT2) and IH [13, 19, 29, 42–52]

Symptom	NT1	NT2	IH
EDS	Required		
Cataplexy	Common	Absent	
Sleep hallucinations	Common	Intermediate	Occasional
Sleep paralysis	Common	Intermediate	Occasional
Disrupted nocturnal sleep	Common	Intermediate	Occasional
Long sleep times	Not typical	Intermediate	Intermediate
Sleep inertia/sleep drunkenness	Occasional	Intermediate	Common
Autonomic symptoms such as orthostatic intolerance	Intermediate		
Memory impairment	Intermediate		
Executive control problems	Intermediate		
Attention deficits	Intermediate		

EDS, excessive daytime sleepiness; IH, idiopathic hypersomnia; NT1, narcolepsy type 1; NT2, narcolepsy type 2.

Colors indicate reported incidence ranges for each symptom, with some overlap due to wide variation among studies. Red: required (95–100%); orange: common (mostly between 40% and 85%); yellow: intermediate (mostly between 20% and 55%); green: occasional (mostly between 5% and 35%); gray: not typical (≤18%) or absent (0%).

ICSD-3 criteria for distinguishing IH from the narcolepsies, particularly NT2, are complex. Although EDS is required for a diagnosis of NT1, NT2, and IH, the ICSD-3 does not address how it varies between them. For example, EDS in NT1 is characterized by daytime sleep attacks, whereas patients with IH rarely experience sleep attacks [29]. Likewise, sleep-related hallucinations and sleep paralysis occur across all three disorders, but have been found to be more common in NT1 than in NT2 or IH. In a French survey study, sleep-related hallucinations occurred in 54%, 30%, and 11% patients, respectively, and sleep paralysis in 45%, 25%, and 11% [42]. In a cross-sectional study in Japan, sleep paralysis occurred in 65%, 32%, and 29% of patients with NT1, NT2, and IH (without long sleep time), respectively, and hypnagogic hallucinations in 70%, 42%, and 33% [54]. Most people with IH have long nocturnal sleep and sleep inertia/drunkenness, in contrast to a lower prevalence in NT2 and especially NT1 (Table 3). In one observational study, 81–84% of untreated individuals with IH exhibited the triad of EDS, long nocturnal sleep, and sleep inertia/drunkenness [7].

**Table 3.** Symptoms associated with IH [1–4, 7, 8, 10, 11, 13, 14, 17, 19, 21, 29, 31, 41–48, 54–102]

Symptoms	Frequency %	Notes
EDS	• 95–100%	• EDS required for definition of IH • ESS scores lower than in NT1, at diagnosis and at follow-up, in some but not all studies
Long (>1 h), unrefreshing naps	• 14–54% require daily naps • 51–83% say naps are unrefreshing	–
Prolonged (>9 or >10 h in 24 h) and undisturbed nocturnal sleep	• 14–58% sleep >10 h at night	• 10-h threshold was used in ICSD-2 and sometimes still is to distinguish sleep duration subtypes • But some patients sleep 9 h during the night and 3 h during the day • Up to a third may have sleep disturbance • Wide variation among studies
Impaired daytime alertness and focus	• 54–84% • Memory deficits in 52–79% • Attention deficits in 59%	• These symptoms are not well characterized in IH
Sleep inertia/sleep drunkenness	• 20–92% • Trouble awakening and functioning with alertness: 40–96% • Requiring multiple alarms: 43–70% • In 12–27%, accompanied by automatic (not consciously directed) behavior, disorientation, confusion, irritability, poor coordination	• Poorly defined • Two studies reported higher frequency of sleep inertia in patients with long sleep times vs. patients without long sleep times
Motor hyperactivity	• NR	• Constantly moving or talking, making to-do lists, quick reactions to questions or requests • Serves as countermeasure to boost alertness
Headaches	• 53–77%	–
Night “blackouts”	• 50–60%	• No memory of anything during sleep
Hallucinations at sleep onset or upon waking	• 4–45%	• Less common than in NT1 • Often occurs alongside sleep paralysis
Sleep paralysis	• 10–42%	• Between wakefulness and sleep, a period of awareness without being able to move, speak or react • Less common than in NT1, but also occurs in 10% of general population • Often occurs alongside sleep-related hallucinations
Vivid dreams/nightmares	• 25–28%	• Percentage reporting nightmares is similar to that in narcolepsy • 20% “tired of dreaming too much” • Rarely studied
Orthostatic hypotension	• 18–43%	–
Autonomic symptoms	• 15–17%	• About 15–17% of IH patients completing the COMPASS-31 questionnaire reported that they had preexisting autonomic disorders
Difficulty with temperature regulation	• 8–25%	• Raynaud phenomenon 25%

EDS, excessive daytime sleepiness; ESS, Epworth Sleepiness Scale; IH, idiopathic hypersomnia; ICSD-2, International Classification of Sleep Disorders, Second Edition; NT1, narcolepsy type 1.

With regard to diagnostic test criteria, there is no difference in mean MSLT sleep latency requirements between IH and NT2, which should not be greater than 8 min for each (Table 1). However, short sleep latency can also occur in the general population [29, 43, 55, 56, 103–105], and although the MSLT has known effects of age, these are not considered when interpreting MSLT results, or for diagnosing these disorders overall [53, 106, 107]. A diagnosis of NT2 requires at least two SOREMPs on the MSLT, compared with zero or one SOREMPs for IH. SOREMPs and sleep latency measurements on the MSLT have low sensitivity and specificity for IH diagnosis and poor test-retest reliability for distinguishing between IH and NT2, allowing for the possibility of misclassification as a result of month-to-month variation [4, 9, 17, 19, 21, 43, 53, 55, 57, 58, 103, 106, 108–121]. Furthermore, there is no relationship of this measure to clinical presentation [9, 53, 59, 122, 123]. In a retrospective database analysis of individuals with a central hypersomnia followed over an average of 4 years, diagnoses changed in half of them as a result of shifting results on MSLT [9]. In a similarly designed study, 26% of the NT2 cases changed from narcolepsy to IH [103]. A Japanese study of 41 individuals with pathological sleep prolongation found that the number of SOREMPs was not significantly related to clinical profile [113]. Objective findings from PSG or actigraphy are often not related to patients’ perception of their own sleep issues, according to results from a prospective observational study conducted in the Netherlands [123].

Findings from studies conducted in Australia, the United Kingdom, and the United States that tested patients’ urine in conjunction with MSLT assessments suggest that undisclosed drug use may affect 10–20% of patients taking sleep tests [124–126]. Amphetamines, cannabinoids, opiates, and benzodiazepines were the most common undisclosed drugs used before testing, and almost none of the patients’ physicians had suspected these agents as a potential cause of the patient’s hypersomnia [124–126]. A simple practical solution is implementation of routine urinary drug screening for all patients undergoing sleep studies [124–126].

Finally, the clinical presentation of IH can be difficult to distinguish from hypersomnia due to psychiatric causes, which is also classified as a CDH by the ICSD-3 criteria. Both IH and psychiatric-related hypersomnia share dysregulated sleep and mood, including long sleep duration and reduced sleep latency, with sleep efficiency similar to healthy controls [44, 127]. In contrast to IH, the degree of EDS does not remain stable over time in patients with mood disorders [21, 44]. The hypersomnia may be a “nonspecific response” to depression [17], or the depression may be a response to the hypersomnia disorder [44, 55, 57, 108]. Furthermore, psychiatric-related hypersomnolence may precede the mood disorder, and it may persist long after the resolution of a depressive episode [17, 128, 129]. Differential diagnosis is further complicated by the tendency of some patients to prefer an IH diagnosis to one that carries a label of psychiatric disease [17].

In addition to diagnostic challenges outlined above, patients may not consider themselves to have a medical problem and may not seek medical advice until such time as their symptoms and experiences have significant impacts on their lives [19]. This, combined with the challenges of diagnosing IH, can lead to considerable delays in diagnosis. Diagnostic delays have been reported by studies conducted in the United States (8 years in adults), the Czech Republic (9 years in adults), South Korea (1 year in adolescents), and India (6 years in adults and children), illustrating the pervasiveness of this issue [10–12, 15].

#### Potential new diagnostic criteria

**Figure 2.**
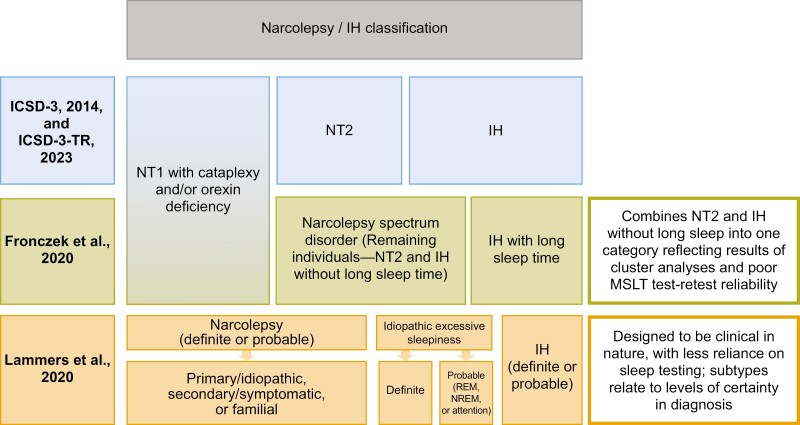
Proposed classification schemes, compared with existing International Classification of Sleep Disorders, Third Edition (ICSD-3), categories [1, 29, 53]. EDS, excessive daytime sleepiness; ICSD-3-TR, International Classification of Sleep Disorders, Third Edition, Text Revision; MSLT, Multiple Sleep Latency Test; NREM, nonrapid eye movement; NT1, narcolepsy type 1; NT2, narcolepsy type 2; REM, rapid eye movement.

Among many improvements to ICSD-3 criteria for IH suggested within the existing published literature, two new classification systems have been proposed (Figure 2) [29, 53].

One system advocated at the Seventh International Symposium on Narcolepsy (2018) restores the distinction between IH with and without long sleep duration [29]. This system combines IH without long sleep and NT2 into a “narcolepsy spectrum disorder” category, because these phenotypes cannot be distinguished using current tests and there is no evidence that they do not share pathophysiology [5, 11, 29, 130]. It reflects results of cluster analyses of individuals with narcolepsy or IH referred to sleep disorder centers, which revealed that people with NT2 or IH without long sleep time formed a single cluster [8]. Correspondingly, after ruling out insufficient sleep syndrome and hypersomnia associated with other comorbidities, the following three categories were presented in their suggested classification:

- NT1 with cataplexy and orexin deficiency;
- IH with long sleep time, with extended sleep over 24-hour periods;
- “Narcolepsy spectrum disorder,” composed of NT2 plus IH without long sleep time [29].

Although this classification offers three distinct and measurable phenotypes [29], cutoffs for nocturnal long sleep time have not been defined [11, 19, 29].

A second classification system was suggested by a European Task Force established to update existing diagnostic criteria for CDHs [53]. This system was designed to be more clinical in nature, with less reliance on sleep testing, and incorporates levels of certainty in diagnosis. The schema contains the following three categories, after ruling out sleep apnea, chronic sleep deprivation, and circadian rhythm disorders:

- Narcolepsy (with subtypes, but not NT1 vs. NT2);
- IH;
- Idiopathic excessive sleepiness (with subtypes) [53].

The system incorporates levels of certainty as follows:

- “Definite” IH can be diagnosed after confirming normal orexin concentration, sleep efficiency > 85% (“at least up to middle age”), presence of excessive need for sleep, the fact that “the ‘excessive need for sleep’ complaint is acquired” and objective evidence for increased sleep need assessed using actigraphy and PSG;
- “Probable” patients with IH meet all but the last criteria, because they only show “objective support” for increased sleep need using PSG and actigraphy (vs. “objective evidence” for “definite” patients with IH) [53].

This system eliminates all hypersomnia entities due to other (medical, substance, or psychiatric) causes, because it is not possible to tell whether these other problems have a causal relationship with hypersomnia or are incidental [17, 53, 131, 132].

A range of other improvements to the existing ICSD-3 diagnostic criteria have been suggested for inclusion in the next version, such as better definitions of sleep efficiency, daytime naps, and sleep inertia/drunkenness [29, 133, 134] with retention of extended PSG to assess long sleep time [29, 135]. Efforts have been made to focus on duration of total sleep time as a way to better distinguish IH [56, 103, 113]. Extended sleep monitoring for >32 hours has also been recommended because sleep durations of ≥19 hours during this span of monitoring best distinguish patients with IH from controls [56]. Alternatively, total sleep time of ≥11 hours over a 24-hour period should indicate IH, regardless of SOREMPs [113]. An ongoing study, the Swiss Primary Hypersomnolence and Narcolepsy Cohort Study, is in the process of investigating the clinical profile of NT1 and the “narcoleptic borderland” to improve future diagnosis [23].

### Natural history of IH

After diagnosis, IH tends to follow a stable and typically lifelong course; however, the natural history of IH is not well studied. In a study of 43 adults with IH diagnosed in the previous 20 years, more patients without long sleep than with long sleep had a stable or improving disease course (83% vs. 61%) [11]. The group with long sleep time was more often intolerant or refractory to standard treatments, which might be a contributing factor to their less favorable disease course [11]. Multiple studies indicate that 11–33% of patients may experience remission [29, 43, 57, 60–62, 136], although in some cases, remissions may be caused by insufficient clarity of diagnosis and may be a result of previous false positive diagnoses. In a longitudinal study conducted in South Korea, 24 individuals with IH and 22 with NT2 had similar remission rates (45% at 5 years post diagnosis in NT2 and 33% at 5.5 years in IH) [136]. None of the 28 patients with NT1 included in the study were in remission [136], supporting the theory that NT1 could be distinguished clinically by its absence of spontaneous remission [29, 43, 57, 136]. It is also plausible that spontaneous remission in NT2 and IH may account for some test–retest discordance on MSLT, although this has not been tested [136].

### Burden of IH

#### Clinical burden

Although narcolepsy is associated with EDS and irresistible daytime naps, people with IH (especially those with long sleep time >10 hours/day) rarely, if ever, have sleep attacks [29, 44–47]. Instead, they have a constant level of drowsiness and “fogginess,” and seldom feel fully alert [29, 46, 47]. They have trouble sustaining attention for more than 1 hour, compared with nearly 4 hours on average in controls [29]. A 2021 systematic review of the literature identified four studies that assessed cognition in IH, and found that patients with IH have longer reaction times and more variable reaction times on vigilance tasks; other cognitive measures were poorly investigated [31]. Patients with IH with severe sleep inertia/sleep drunkenness feel “foggiest” when they wake up, and better as the day progresses [19, 137]. Sleep does not refresh patients with IH as it does for those with NT1 [47, 55]. Individuals with IH have been termed “night owls” because they can focus best in the evening [19, 47, 55, 63].

Notably, fatigue was rarely reported among symptom profiles in IH, possibly owing to overlap with EDS and its lack of specificity for IH [17, 138]. Fatigue scores were similar between patients with IH and those with subjective EDS in a large US series of 187 hypersomnolent patients, 63 of whom had IH [138], and between individuals with IH and NT1 in a Swedish study [16]. In the Swedish study, Fatigue Severity Scale scores were significantly higher for IH than for healthy controls, indicating high fatigue burden in IH [16]. In an online survey of 138 people with self-reported IH and 81 matched controls recruited through the website of a US patient advocacy group, patients with IH reported more fatigue than controls. However, an additional control group of 24 investigator-recruited patients with confirmed IH did not report excess fatigue [63].

Severity of IH symptoms has been shown to vary considerably between patients. On the Idiopathic Hypersomnia Severity Scale (IHSS), 5% of participants were classified as mild, 22% moderate, 53% severe, and 19% very severe. Women and people reporting long nocturnal sleep were more likely to be rated as severe/very severe [7].

#### Comorbidities

There are several comorbidities associated with IH, including autoimmune, autoinflammatory, and allergic disorders [139], systemic exertion intolerance disease [63, 138], postural orthostatic tachycardia syndrome [63], obesity [139, 140], migraine [64], attention deficit and hyperactivity disorder [141, 142], and depression and anxiety [7, 11, 14, 16, 17, 42, 64–66, 106, 113, 139, 143–146]. It is possible that some of these comorbidities may represent misdiagnoses, some may be aggravating factors in the development and maintenance of IH, and others may have diagnostic overlap with IH. The most thoroughly studied and commonly reported comorbidities of IH are mood disorders, specifically depression and anxiety (Table 4). These are considered comorbidities when ICSD-3 is used for diagnosis, but not when DSM-5 is used.

**Table 4.** Studies reporting on depression in people with IH

Citation	Country	No. of patients with IH	Patients with depression (%) or mean/median score	Definition of depression used
Barateau et al., 2016 [143]	France	91 (89 with BDI score)	22.5% (20/89)	BDI > 19
Barateau et al., 2017 [139]	France	138	19.57% (27/138)	BDI > 19
Bušková et al., 2021 [17]	Czech Republic	15	47%	BDI-II ≥ 20
Cairns and Bogan, 2019 [106]	United States	25	18%	“Diagnosed depression”
Chabani et al., 2020 [65]	France	133	Median (IQR): 6 (3–9)	HADS (score 0–21)
Dauvilliers et al., 2019 [58]	France	102 (59 untreated and 43 treated)	Mean for 59 untreated: 14.55 ± 9.42	BDI-II
Evangelista et al., 2022 [66]	France	62 (35 with severe sleep inertia in the last month, “M-sleep inertia”; 44 with long sleep inertia the day of the PVT, “D-sleep inertia”)	M-sleep inertia group: 32.35% (11/34) D-sleep inertia group: 29.55% (13/44)	BDI-II ≥ 20
Honda et al., 2022 [113]	Japan	41 patients with “‘pathological sleep prolongation’” (24-h PSG TST ≥ 660min), of whom 6 had ≥2 SOREMPs and 35 ≤ 1 SOREMP on the MSLT	39% (16/41)	PHQ-2 ≥ 3
Leu-Semenescu et al., 2022 [42]	France	221	20.7%	BDI-II > 19
Lopez et al., 2017 [108]	France	20	31.6% (6/19)	“Depressive symptomatology”
Nevsimalova et al., 2021 [11]	Czech Republic	25 with normal sleep and 18 with long sleep duration	Mean ± SD: normal sleep, 6.28 ± 6.8; long sleep, 8.7 ± 6.3	BDI
Ong et al., 2020 [145]	United States	12 (inclusion criteria was PHQ-8 ≥ 5)	Mean ± SD: baseline, 14.42 ± 3.73; post treatment (CBT-H): mean ± SD: 11.75 ± 4.59	PHQ-8 (PHQ-9 without item 9, suicidal ideation)
Pascoe et al., 2019 [146]	United States	39	58.3% (21/36); 35.9% (14/39)	PHQ-9 ≥ 10; “comorbid depression”
Rassu et al., 2022 [7]	France	Cross-sectional sample: 166 untreated and 60 treated; longitudinal sample: 77 before and after treatment	Cross-sectional sample: 23.23% (36/155) and 13.46% (7/52); longitudinal sample: 33.33% (14/42) and 16.67% (7/42)	BDI-II ≥ 20
Sowa et al., 2016 [14]	No restrictions on geography	235	15.1%	“Depression”
Suzuki et al., 2015 [64]	Japan	35	Mean ± SD: 10.6 ± 7.8	BDI-II
Wasling et al., 2020 [16]	Sweden	21	28% (6/21)	PHQ-9 ≥ 10

BDI, Beck Depression Inventory; BDI-II, Beck Depression Inventory-II; CBT-H, Cognitive Behavioral Therapy for Hypersomnia; HADS, Hospital Anxiety and Depression Scale; IH, idiopathic hypersomnia; IQR, interquartile range; MSLT, Multiple Sleep Latency Test; PHQ-2, Patient Health Questionnaire-2; PHQ-8, Patient Health Questionnaire-8; PHQ-9, Patient Health Questionnaire-9; PSG, polysomnography; PVT, Psychomotor Vigilance Test; SOREMP, sleep-onset rapid eye movement period; TST, total sleep time.

People with more severe IH may have a higher risk of comorbid depression, although IH remission does not appear to affect depression rates. Individuals with IH who scored higher on the IHSS had a higher rate of depressive symptoms on the BDI; of those with IHSS scores in the most severe of four severity categories, 40% had moderate/severe depression (BDI of 20 and above) [7]. The presence of moderate/severe depression was not related to the presence or absence of drug treatment for IH [7] or to IH remission [136].

Depression has been reported in 13–36% of people with IH in multiple studies using various definitions and across a variety of geographical regions, in comparison with 4–5% of healthy controls [7, 14, 16, 42, 136, 139, 146]. Although most studies are in adults, depression has also been reported in pediatric populations [12]. In an internet survey in the United States, of 371 people with self-reported hypersomnia disorders (two-thirds narcolepsy, one-third IH), 61–91% responded that they had symptoms of depression and anxiety, including sad mood, loss of interest, irritability, social isolation, concentration deficits, feelings of guilt or worthlessness, anxiety, or worry [13].

It remains unclear whether depression is more or less common in IH than in narcolepsy or other CDHs [12, 13, 42, 67, 139, 146]. There was no difference between depression scores among 15 people with IH and 52 with psychiatric-related hypersomnia in a study conducted in the Czech Republic [17]. In contrast, a South Korea study reported a significantly higher rate of depressive symptoms in people with IH (25%) than with NT1 (3%) or NT2 (9%); the rate of comorbid depression in the IH cohort was similar to that of a group of 33 people with subjective hypersomnia (30%) [136].

Survey data indicate that the presence of depression does not seem to change hypersomnia symptoms materially [10]. Only two symptoms differed: the need for multiple alarms to wake up (67% with depression vs. 75% without), and the need for intentional naps within the last 30 days while receiving treatment for IH (19% vs. 9%) [10].

In contrast to depression, few studies have assessed the presence of anxiety in patients with IH. One small US study reported anxiety in 9 of 39 patients with IH (23%), a prevalence that did not differ significantly by CDH type [146].

#### Humanistic burden

Negative effects of IH have been reported across multiple domains of health-related quality of life (HRQOL), productivity, and health-state utility scores (measures of patient preferences for particular health states) in affected patients (Table 5). The European Quality of Life–5 Dimensions Questionnaire and Short Form-36 were the most commonly used instruments assessing HRQOL and health status in IH studies. Lower HRQOL scores than in healthy controls were observed in most domains of these instruments; role physical and role emotional were the most affected and physical function and pain were the least affected [14, 63, 68, 149–151]. The magnitude of effects on HRQOL are similar for IH and narcolepsy in both adults and children and in individuals from a variety of countries and regions [16, 145, 151–162].

**Table 5.** HRQOL and utility outcomes in IH populations

Author, year	Country	Study design	Population	HRQOL and utility outcomes
Dauvilliers et al., 2019 [58]	France	Cross-sectional cohort	• 102 IH (59 untreated, 43 treated)	**EQ-5D utility** (mean ± SD): 0.77 ± 0.20 **EQ-5D VAS** (mean ± SD): 63.61 ± 21.47
Miglis et al., 2020 [63]	United States	Prospective cohort	• 138 online IH • 24 confirmed IH • 81 matched controls	**RAND-36** (total score NR) All scores for online patients were significantly lower than online controls (*p* < .001): Scores for online patients, confirmed patients, and online controls, respectively, mean (IQR): • Physical functioning: 65 (45–80), 80 (58–95), 90 (80–95) • Role physical: 25 (0–25), 75 (25–100), 100 (75–100) • Role emotional: 50 (0–100), 100 (50–100), 100 (75–100) • Energy/fatigue: 10 (5–20), 50 (15–56), 55 (40–65) • Emotional well-being: 60 (44–76), 66 (60–80), 72 (60–80) • Social functioning: 44 (25–63), 75 (50–100), 75 (63–100) • Pain: 68 (45–90), 85 (68–90), 90 (68–90) • General health: 35 (25–60), 60 (38–79), 70 (60–80)
Nevsimalova et al., 2021 [11]	Czech Republic	Prospective cohort	• 25 IH without long sleep duration and 18 with long sleep duration treated or followed for ≥ 1 year	**SF-36** total scores (domain scores NR), comparison NS: • Long-sleep IH (mean ± SD): 7.5 ± 2.1 • Without long sleep IH: 6.6 ± 1.7
Ong et al., 2020 [145]	United States	Clinical trial	• 12 IH with depression assigned to individual vs. group delivery of online CBT	**PROMIS**: baseline; post treatment (mean ± SD), both comparisons NS: • Global mental health: 37.74 ± 4.96; 37.19 ± 7.61 • Global physical health: 42.53 ± 7.92; 42.13 ± 4.30
Ozaki et al., 2012 [68]	Japan	Prospective cohort	• 54 IH without long sleep time treated for EDS with ≥ 1 year of follow-up • 82 drug-naïve IH historical controls	**SF-36** (total score NR), treated; drug-naïve; *p-*value for comparison (mean ± SD); *p*-value for comparison with national normative data: • Physical health: 52.9 ± 6.1; 50.5 ± 9.7; NS; *p = *.001 • Role physical: 40.2 ± 6.9; 36.2 ± 23.6; NS; *p < *.001 • Bodily pain: 49.4 ± 10.2; 49.4 ± 12.0; NS; NS • General health: 45.5 ± 9.3; 46.5 ± 10.9; NS; *p < *.001 • Vitality: 44.4 ± 10.0; 43.1 ± 9.9; NS; *p < *.001 • Social functioning: 47.7 ± 11.0; 43.7 ± 12.9; NS; NS • Role emotional: 43.7 ± 5.1; 36.4 ± 21.7; *p = *.004; *p < *.001 • Mental health: 50.1 ± 7.9; 44.2 ± 10.2; *p < *.001; NS
Rassu et al., 2022 [7]	France	Prospective and cross-sectional	• Cross-sectional sample: 166 untreated and 60 treated IH • Longitudinal sample: 77 of the untreated patients who were then treated	Among numbers of patients with available values (*n*): • **EQ-5D utility** score (mean ± SD): ◦ Cross-sectional sample (*p = *.20) • Untreated (*n = *145) 0.77 ± 0.20 • Treated (*n = *50) 0.80 ± 0.18 ◦ Longitudinal sample (*p = *.05) • Untreated (*n = *39) 0.73 ± 0.19 • Treated (*n = *39) 0.78 ± 0.19 • **EQ-5D VAS**: ◦ Cross-sectional sample (*p = *.03) • Untreated (*n = *142) 61.96 ± 19.47 • Treated (*n = *54) 68.26 ± 18.75 ◦ Longitudinal sample (*p = *.14) • Untreated (*n = *57) 59.58 ± 22.10 • Treated (n = 57) 62.86 ± 18.24
Trotti et al., 2015 [147]	United States	Randomized controlled trial	• 5 IH with long sleep • 5 IH without long sleep	**SF-36** differences between clarithromycin and placebo: • Results from original published study on SF-36 energy (mean ± SD): ◦ Long sleep: 22.3 ± 21.3 ◦ Without long sleep: 6.0 ± 11.8 • Previously unpublished total SF-36 score reported in the 2021 systematic literature review considered improvements “clinically” but not statistically significant: mean difference (95% CI) 9.70 (−1.63 to 21.03) [148] • 2021 Cochrane review: using these previously unpublished SF-36 score data from 6 of the 10 patients with IH, no differences were found on any of the SF-36 subscales [35]
Wasling et al., 2020 [16]	Sweden	Prospective and cross-sectional	• 21 IH • 23 healthy controls	• **EQ-5D-5L index** (mean ± SD), 0.835 ± 0.174; significantly lower than controls (*p < *.0001) • **EQ-5D VAS**, 64.4 ± 21.8; significantly lower than controls (*p < *.0001) • **Significant determinants of (EQ-5D-5L index, VAS)** in IH: ◦ PHQ-9 (0.64 [*p < *.0001], 0.50 [*p = *.0003]) ◦ FSS (0.24 [*p = *.03], 0.38 [*p = *.005]) ◦ Rate of employment or studies (0.18 [*p < *.0001], 0.33 [*p = *.006]) ◦ Rate of activity or sickness compensation (0.64 [*p < *.0001], 0.51 [*p = *.0003]) • **No significant effect** from sleep latency, age, age at symptom onset, disease duration, ESS, BMI, CSF orexin-A, or procrastination scales

BMI, body mass index; CBT, cognitive behavioral therapy; CSF, cerebrospinal fluid; EDS, excessive daytime sleepiness; EQ-5D, European Quality of Life–5 Dimensions Questionnaire; FSS, fatigue severity scale; HRQOL, health-related quality of life; IH, idiopathic hypersomnia; IQR, interquartile range; NR, not reported; NS, not significant; PHQ-9, Patient Health Questionnaire-9; PROMIS, Patient-Reported Outcomes Measurement Information System; SF-36, Short Form-36; VAS, Visual Analog Scale.

Conflicts in social, work, and family life because of unusual sleep needs may be responsible for lowering HRQOL [16, 44, 63, 68, 150, 152, 162–164]. People often feel guilt and social anxiety over the diagnosis, its symptoms, and resulting functional limitations [165]. These issues may also contribute to depressive symptoms, which in some studies are the major, but not the only, determinant of HRQOL [16, 67, 68, 144, 151, 154, 157, 166–168]. Subjective sleepiness may be only indirectly associated with the factors influencing HRQOL, as in some studies it has no significant connection to HRQOL domains in drug-naïve patients [68, 150].

Utility scores in IH ranged from 0.73 to 0.84 in two studies conducted in France and one conducted in Sweden, all using the European Quality of Life–5 Dimensions Questionnaire (Table 5) [7, 16, 58]. The two French studies found no significant differences in utility scores between IH and NT1 [7, 58], whereas in Sweden, utility scores in patients with IH (0.84) were lower than those in healthy controls (0.95) [16]. Utility scores in IH and in NT1 in Sweden were comparable with those reported in France for people with hemophilia (0.74) [169].

IH has a disabling effect on several major areas of daily function, including school and work performance, ability to interact with family and friends, driving ability, and pedestrian safety (Table 6). Often, people with IH are late to school or work in the morning because of difficulty in waking. They need help from another person to do so, creating unwanted dependence [7, 17, 19, 47, 174]. At work, people with IH may lose their job because they are late [19, 47, 174]. Job loss, change in employment, and career interference have been reported as problems by more than a third of people with hypersomnia, potentially curtailing their income [68, 154, 157, 175, 176].

**Table 6.** Functional outcomes in IH populations

Author(s), year	Country	Study design	Population	Functional outcomes
Bijlenga et al., 2021 [170]	The Netherlands	Prospective cohort	• 6 IH	3 of 6 patients with IH were found to be **at an increased risk of impaired driving**
Dauvilliers et al., 2022 [171]	Belgium, Czech Republic, Finland, France, Poland, Spain, USA	Randomized controlled trial	115 IH: • 56 LSO • 59 placebo	• **WPAI:SHP**: improvements with LSO vs. placebo on work productivity • **FOSQ-10** score significantly improved with LSO. Estimated median difference (95% CI) between LSO and placebo groups in change in FOSQ-10 score from the end of the stable-dose period to the end of the double-blind, randomized withdrawal period: 3.7 (2.5–5.0); *p < *.0001
Mayer et al., 2015 [69]	Germany	Randomized controlled trial	IH drug-free without long sleep: • 14 placebo • 17 modafinil	**6-point scale of effectiveness/performance**: significant improvements with modafinil vs. placebo by week 3: mean difference (95% CI) −0.68 (−1.26, −0.10); *p = *.033
Ong et al., 2020 [145]	USA	Clinical trial	12 IH with depression who participated in online CBT	• **PROMIS**: baseline; post treatment (mean [SD]), both comparisons NS: ◦ Global mental health: 37.74 [4.96]; 37.19 [7.61] ◦ Global physical health: 42.53 [7.92]; 42.13 [4.30] • **FOSQ**: baseline 2.21 [0.39]; post treatment 2.33 [0.46]; * p = *0.2688
Ozaki et al., 2012 [68]	Japan	Prospective cohort	• 54 IH without long sleep time treated for EDS with ≥ 1 year of follow-up • 82 drug-naïve IH historical controls	**Sociodemographic variables** of treated patients with IH: • Experience of divorce or break up with partner due to symptoms: yes, 13.0%; no, 87.0% • Experience of being forced to relocate or being dismissed due to symptoms: yes, 25.9%; no, 74.1%
Philip P et al., 2014 [172]	France	Randomized controlled trial	• 14 IH who crossed over between modafinil and placebo • 13 narcolepsy • 14 healthy controls	Assessment of driving performance in patients with narcolepsy and IH (mean ± SD): • Driving performance for narcolepsy vs. IH, respectively: ◦ **Inappropriate road line crossings** over 230 km: 2.0 ± 0.7 vs. 1.3 ± 0.6; *p = *NS ◦ **SD of lateral position of the vehicle:** 25.0** **± 1.1 vs. 23.5 ± 1.0 cm; *p = *NS • Driving performance for narcolepsy/IH vs. controls, respectively (placebo): ◦ **Inappropriate road line crossings** over 230 km: 2.1 ± 0.5 vs. 0.2 ± 0.7; *p < *.05 • Driving performance for narcolepsy/IH vs. controls, respectively (modafinil): ◦ **Inappropriate road line crossings** over 230 km: 1.1 ± 0.2 vs. 0.2 ± 0.7; *p < *.05 • Driving performance for narcolepsy/patients with IH treated with modafinil vs. placebo, respectively: ◦ **SD of lateral position of the vehicle:** 23.6 ± 0.6 vs. 24.9 ± 0.9; *p = *.06
Pizza et al., 2015 [114]	France	Cross-sectional study	• 71 IH • 129 NT1 • 82 NT2 • 470 healthy controls	**Risk of a driving accident in the last 5 years** in IH: • Vs. healthy controls: ◦ Adjusted for sex, age, marital status, coffee, and energy drink intake: OR (95% CI) 2.04 (1.05–3.95) ◦ Adjusted for previous covariates plus ESS and naps: OR 2.31 (0.94–5.71) • Vs. patients with NT1: ◦ Adjusted for sex, age, civil status, energy drink consumption, and disease duration: OR 1.37 (0.63–3.00)
Rassu et al., 2022 [7]	France	Prospective and cross-sectional	• Cross-sectional sample: 166 untreated and 60 treated IH • Longitudinal sample: 77 of the untreated patients who were then treated	Among numbers of patients with available values (*n*), **“significant/very significant” impact of hypersomnolence on driving performance**: • Cross-sectional sample; *p = *.0006 ◦ Untreated (*n = *166) 64 (38.55%) ◦ Treated (*n = *60) 8 (13.33%) • Longitudinal sample; *p < *.0001 ◦ Untreated (*n = *77) 40 (51.95%) ◦ Treated (*n = *77) 20 (25.97%)
Trotti et al., 2015 [147]	United States	Randomized controlled trial	• 5 IH with long sleep • 5 IH without long sleep	**FOSQ** differences between clarithromycin and placebo: • Results from original published study on FOSQ (mean ± SD): ◦ Long sleep: 3.5 ± 3.7 ◦ Without long sleep: 0.3 ± 0.9 • Previously unpublished total SF-36 score reported in the 2021 systematic literature review by Maski et al. [33]: Maski et al. considered improvements “clinically” but not statistically significant: mean (95% CI) 1.9 (−0.5 to 4.3) • Trotti et al., 2021 Cochrane review [35]: using these previously unpublished FOSQ score data from 6 of the 10 patients with IH, no significant difference was found: 0.79 (−3.02, 4.60)
Van Schie et al., 2012 [173]	The Netherlands	Prospective cross-sectional	• 42 NT1 • 5 NT2 • 12 OSA • 37 IH without long sleep	**SART** **accuracy** • No significant difference in error score/225 between NT1, NT2, OSA, and IH groups, respectively: ◦ Median (IQR) 11.1 (6.0–17.4), 10.8 (9.5–21.1), 8.4 (6.1–14.3), and 9.0 (5.9–16.3); *p > *.64 **SART mean RT**: • No significant difference in mean RT between NT1, NT2, OSA, and IH groups, respectively: ◦ Mean ± SD: 337 ± 83 ms, 332 ± 74 ms, 366 ± 87 ms, and 359 ± 82 ms; *p > *.55 • No correlation of SART with MSLT
Wasling et al., 2020 [16]	Sweden	Prospective and cross-sectional	• 21 IH • 23 healthy controls	• **Procrastination scales:** patients with IH scored significantly higher than healthy controls on IPS, but not on PPS or STS • **Domains of EQ-5D-5L**: no difference from controls in mobility or self-care, but significantly worse on usual activities

CBT, cognitive behavioral therapy; EDS, excessive daytime sleepiness; EQ-5D, European Quality of Life-5 Dimensions Questionnaire; ESS, Epworth Sleepiness Scale; FOSQ, Functional Outcomes of Sleep Questionnaire; IH, idiopathic hypersomnia; IPS, Irrational Procrastination Scale; LSO, lower-sodium oxybate; MSLT, Multiple Sleep Latency Test; NS, not significant; NT1, narcolepsy type 1; NT2, narcolepsy type 2; OR, odds ratio; OSA, obstructive sleep apnea; PPS, Pure Procrastination Scale; PROMIS, Patient-Reported Outcomes Measurement Information System; RT, reaction time; SART, Sustained Attention to Response Task; SF-36, Short Form-36; STS, Susceptibility to Temptation Scale; WPAI:SHP, Work Productivity and Activity Impairment Questionnaire: Specific Health Problem.

An understudied effect of IH on school and work is the possibility of diminished cognitive functioning. Self-reports by patients indicate that they believe that IH has an important effect on their intellectual function, meriting further research [7]; however, the effects of any intellectual dysfunction on school and work resulting from IH remains an important knowledge gap [10, 32].

As in narcolepsy, people with IH often have a higher than normal incidence of car accidents, along with other types of accidents, such as workplace injuries [30, 44, 57, 114, 134, 150, 152, 164, 172, 177–185]. Notably, there is a large variation in driving performance among individuals with CDHs, but not across the CDH diagnoses, which are often indistinguishable in terms of degree of driving impairment [114, 172, 179, 181, 185, 186]. Driving impairment may result from problems with vigilance or attention, rather than sleepiness [170, 172, 178, 187–190]. Many countries have responded to the danger posed by sleep-related accidents by regulating driver’s licenses for people with hypersomnia disorders, especially for those who drive professionally.

IH is associated with deleterious effects on many aspects of daytime functioning and HRQOL, and the extent of these detriments may relate to EDS [13, 63, 68]. Many affected patients are forced to move or divorce/break up with a partner for reasons related to their symptoms [68]. People with IH, especially those with poor health status on an HRQOL instrument, report that daily tasks are substantially affected by their IH [7]. Adults with IH are also concerned about the social stigma of sleepiness [165, 191, 192], and some expressed relief at the social avoidance mandated by coronavirus disease 2019 (COVID) lockdowns in France [175]. No studies meeting the criteria for inclusion in the current systematic literature review were identified that pertained to caregiver burden in IH.

#### Treatment

Treatment, typically stimulants developed for narcolepsy, does not fully resolve symptoms, while the effect of many treatments on quality of life and function has not been widely studied [7, 10, 68, 146]. A survey study in Japan of 54 people with IH without long sleep time found that after taking psychostimulants for at least 1 year, their Epworth Sleepiness Scale score significantly improved [68]. A cross-sectional study in France of 166 untreated and 60 treated individuals (median 0.9 years) with IH found that the treated patients had a favorable lower total score on the IHSS [7]. Similarly, a US study assessing sleep-related patient-reported outcomes with pharmacotherapy in patients with IH found that although scores on sleep-related instruments improved, EDS and other symptoms persisted even after 6–24 months of treatment [146]. Cross-sectional survey data indicate that up to two-thirds of treated respondents, most of whom reported having IH, reported persistent daily EDS, difficulty awakening even with multiple alarms, problems functioning with normal alertness, cognitive symptoms, and memory deficits despite current treatment (defined as treatment administered within the previous 30 days) [10]. The number of residual symptoms in self-reported IH was similar to self-reported NT1, but higher than that in NT2 [10].

Only one pharmaceutical agent is approved in the United States for IH—calcium, magnesium, potassium, and sodium oxybate (lower-sodium oxybate)—which was shown to improve function in daily activities and work productivity relative to placebo in a phase 3, double-blind, randomized, withdrawal study in which all participants received lower-sodium oxybate in an initial open-label period prior to randomization to continued lower-sodium oxybate or placebo [171]. At the end of a 2-week, double-blind, randomized withdrawal period, 88% of patients randomized to placebo reported worsening on the Patient Global Impression of Change relative to the end of the stable-dose lower-sodium oxybate period, 5% reported no change, and 7% reported improvement. Conversely, for the patients randomized to the lower-sodium oxybate continued use arm, the percentages were 21%, 41%, and 37%, respectively [171]. Treatments that target IH symptoms can improve some measures of function, such as driving and daily tasks, but often does not restore function to the level of controls without EDS, although patients receiving long-term treatment may see restoration of driving ability closer to controls [7, 114, 170, 172, 185]. Other medications used for narcolepsy, e.g. modafinil, pitolisant, mazindol, pemoline, and clarithromycin, have been found to offer benefits to some patients with IH when used off-label for the treatment of EDS [3, 10, 11, 19, 193]. Modafinil, the most thoroughly studied compound for IH, was found to improve measures of wakefulness and sleepiness (the maintenance of wakefulness test and Epworth Sleepiness Scale score) compared to placebo [45, 69, 163], and improved driving performance and general effectiveness versus placebo [172], although a significant burden remains. A meta-analysis concluded that there was insufficient evidence to determine whether other narcolepsy medications are effective for the treatment of IH [35].

## Discussion

By systematically evaluating clinical, interventional, humanistic, and economic publications relevant to IH up to February 2022, we have identified strong evidence that IH is a poorly defined diagnosis of exclusion and its burden extends beyond excessive sleepiness. In IH, EDS tends to manifest as daytime cognitive dysfunction with impaired alertness, and most patients with IH also have sleep inertia/drunkenness and long nocturnal sleep. However, symptom prevalence is difficult to estimate due to poorly defined diagnostic criteria for IH and symptom overlap among CDHs [7].

Current diagnostic criteria for IH are regarded as an obstacle to research, diagnosis, and disease management. ICSD-3 criteria are dissociated from clinical profiles, which may contribute to routine diagnostic delays [1, 10–12, 15, 18], while the recently updated ICSD-3-TR [38] does little to improve the diagnosis of IH or its differentiation from NT2 owing to very few changes relevant to either condition. A lack of efficacious treatments for IH may also reduce a physician’s motivation to diagnose. Easy-to-use, highly protocolized diagnostic criteria are needed that include measures aligned with clinical presentation. Changes in the definition of IH would allow characterization of IH epidemiology, which has knowledge gaps under the current classification. For example, sleep duration subtypes, omitted from ICSD-3 and the ICSD3-TR, could be re-incorporated into future proposed systems [1]. About half of patients with IH have long overnight sleep times >10 hours and consensus is needed on a threshold for long nocturnal sleep based on objective levels of evidence [10]. Further, most studies have been conducted in adults with IH, and pediatric data on IH are limited; it is plausible that different diagnostic criteria may be needed for children.

The burden of disease for individuals with IH was shown to be high; we found that patients have diminished HRQOL, productivity and utility outcomes, with similar impacts as reported for narcolepsy, although no studies reporting on healthcare costs or healthcare resource utilization were identified for IH. The main driver of reduced HRQOL in IH is likely to be impaired function as a consequence of excessive sleep and unusual sleep patterns, as evidenced by impairments in cognitive functioning, school and work performance, driving ability, and difficulty with interactions with friends and family. Individuals with IH had a high prevalence of comorbid depression, which is likely to contribute to impaired HRQOL. An etiologic or pathophysiologic association between IH and some comorbidities cannot be ruled out, and some comorbidities could contribute to misdiagnoses.

Although the natural history of IH remains poorly understood, IH severity and phenotype are known to vary over time [3, 6, 19, 20, 70, 194]. Findings from this systematic review revealed that patients with long overnight sleep times were less likely to show improved or stable disease course [11], more likely to suffer from sleep inertia [7], and to be less tolerant or refractory to treatments [11], than those without long sleep times. It remains unclear whether cases of spontaneous remission observed in patients with IH may be a result of prior misdiagnosis.

The lifelong course of disease translates into a requirement for lifelong treatment. Lower-sodium oxybate has been shown to improve symptoms and function in patients with IH, although not to the level of healthy controls; however, there is a lack of long-term data with this agent in patients with IH. Further, there are few alternative pharmacological treatment options approved for the treatment of IH for patients not responding to treatment, and non-pharmacological therapy use is high. Since the synthesis of results from this literature review, additional articles on the burden of IH have been published, with findings consistent with the results from our systematic review. The US-based ARISE real world IH hypersomnia outcomes study found that participants with IH had substantial symptom burden and low treatment satisfaction with the mostly off-label and non-pharmacological treatments used to manage their symptoms [195], while a survey study conducted between March and May 2020 found that the COVID-19 lockdown restrictions had significant effects on sleep pattern and duration in patients with sleep disorders including IH [196].

The systematic literature review was performed according to PRISMA guidelines; however, as with all systematic reviews, the overall quality of findings is dependent on the quality of each of the included studies. Critical evaluation of identified studies and study results was conducted, and those with Oxford Levels of Evidence grades 1–3 (highest levels of evidence) were included to ensure certainty of results. A further potential limitation is the small number of identified studies relating to the post diagnosis burden of IH, restricting interpretation of these results to the time of diagnosis for this condition.

Improved understanding of the underlying pathophysiology of the disorders may lead to the identification and development of validated biomarkers of IH and better discrimination between narcolepsy and IH, leading to the development of better therapeutic options. Examples of areas of research into the potential causes of IH include evaluations of the underlying neurobiology of patients with IH [197, 198], investigation of disturbed regulation of circadian rhythms [199], home-based sleep monitoring to assess ad libitum sleep patterns [200], cluster analyses to help define and distinguish disease phenotypes [201], and evaluation of regional brain metabolism to distinguish NT1 and IH [202]. In adults, EEG evidence of a decreased homeostatic drive to sleep was observed in people with IH [203], and analysis of electroencephalographic (EEG)- and PSG-derived metrics, including analysis of EEG activity and sleep microarchitecture, as potential biomarkers for IH [111, 204, 205], have provided insights into the distinction between IH and NT2. However, none have showed promise for definitively distinguishing IH and NT2 to date.

## Conclusions

Overall, this systematic review of literature covering a 10-year period from 2012 to 2022 provides a comprehensive overview of the burden of IH. Despite recent updates to the ICSD-3 criteria for the classification of CDHs, IH remains a poorly defined diagnosis of exclusion that is difficult to distinguish from NT2, while overlapping symptoms and disutility of objective tests contribute to substantial diagnostic delays that add to the overall patient burden. Pharmacological treatment options are limited and do not fully resolve symptoms. We identified significant effects of IH on daily functioning, work productivity, and overall quality of life that continue after treatment initiation. Further collaboration is required to identify and validate objective markers of IH with which the classification of IH and IH subtypes can be reevaluated. This would move IH into a position that could benefit from future targeted interventions.

## Supplementary Material

zpae059_suppl_Supplementary_Material

## Data Availability

The datasets will be made available after the publication of study results within 3 months from initial request to researchers who provide a methodologically sound proposal.
