# Supplementary material for: Diagnostic challenges and burden of idiopathic hypersomnia: a systematic literature review
Source: Sleep Adv. 2024 Aug 16;5(1):zpae059. doi: 10.1093/sleepadvances/zpae059 (PMC11359170; doi:10.1093/sleepadvances/zpae059)
Supplement: zpae059_suppl_Supplementary_Material [file zpae059_suppl_supplementary_material.docx]

**SUPPLEMENTARY APPENDIX TO:**

**Diagnostic challenges and burden of idiopathic hypersomnia: a systematic literature review**

Talia Boulanger^1^, Pascale Pigeon^1^ and Stephen Crawford^2,*^

1. Talia Boulanger, LLC, Westford, MA 01886, USA and
2. Takeda Development Center Americas, Cambridge, MA 02139, USA

*Corresponding author. Stephen Crawford, Takeda Development Center Americas, Cambridge, MA 02139, USA. Email: stephen.crawford@takeda.com.

**Table S1.** Medline (PubMed) search.

| **Purpose** | **Search** | **Query** | **Items found February 17, 2022** |
| --- | --- | --- | --- |
| Find and eliminate old narrative reviews while keeping new ones and systematic reviews/ meta-analyses | 16 | #14 NOT #15 | **167** |
|  | 15 | review[pt] AND 2012:2019[dp] NOT (cochrane OR systematic OR meta-analy*) | 774,941 |
| Find and eliminate case reports | 14 | #12 NOT #13 | 194 |
|  | 13 | case reports[pt] | 2,248,031 |
| Limit to articles with abstracts from the last 10 years | 12 | #8 NOT #9, Filters: Abstract, last 10 years | 206 |
| Limit to articles from the last 10 years | 11 | #8 NOT #9, Filters: Last 10 years | 221 |
| Find and eliminate articles about animals | 10 | #8 NOT #9 | 371 |
|  | 9 | #3 OR #6 OR #7, Filters: Other Animals | 13 |
| Find articles about idiopathic hypersomnia | 8 | #3 OR #6 OR #7 | 384 |
|  | 7 | "primary hypersomnia"[tiab] | 14 |
|  | 6 | #4 OR #5 | 142 |
|  | 5 | "idiopathic hypersomnolence"[tiab] | 12 |
|  | 4 | hypersomnolence, idiopathic[MeSH Terms] | 133 |
|  | 3 | #1 OR #2 | 369 |
|  | 2 | "idiopathic hypersomnia"[tiab] | 354 |
|  | 1 | hypersomnia, idiopathic[MeSH Terms] | 133 |

**Table S2.** Embase search.

| **Purpose** | **Search** | **Query** | **Items found February 18, 2022** |
| --- | --- | --- | --- |
| Limit to articles indexed in Embase | 19 | #18 AND [embase]/lim | 240 |
| Find and eliminate old conference abstracts | 18 | #16 NOT #17 | 258 |
|  | 17 | 'conference abstract'/it AND [2010-2019]/py | 3619252 |
| Find and eliminate old narrative reviews while keeping new ones and systematic reviews/ meta-analyses | 16 | #14 NOT #15 | 443 |
|  | 15 | 'review'/it AND [2012-2019]/py NOT (cochrane OR systematic OR 'meta analy*') | 724025 |
| Find and eliminate case reports | 14 | #12 NOT #13 | 480 |
|  | 13 | 'case report' | 2844120 |
| Limit to articles with abstracts from the last 10 years | 12 | #11 AND [abstracts]/lim | 536 |
| Limit to articles from the last 10 years | 11 | #10 AND [2012-2022]/py | 553 |
| Find and eliminate articles about animals | 10 | #8 NOT #9 | 855 |
|  | 9 | #8 AND ([animal cell]/lim OR [animal experiment]/lim OR [animal model]/lim OR [animal tissue]/lim) | 8 |
| Find articles about idiopathic hypersomnia | 8 | #3 OR #6 OR #7 | 863 |
|  | 7 | 'primary hypersomnia':ti,ab | 36 |
|  | 6 | #4 OR #5 | 175 |
|  | 5 | 'idiopathic hypersomnolence':ti,ab | 24 |
|  | 4 | 'somnolence'/exp AND 'idiopathic disease'/de | 151 |
|  | 3 | #1 OR #2 | 706 |
|  | 2 | 'idiopathic hypersomnia':ti,ab | 676 |
|  | 1 | 'hypersomnia'/de AND 'idiopathic disease'/de | 120 |

**Table S3.** Study inclusion/exclusion criteria.

| **Included** | **Excluded** |
| --- | --- |
| Articles reporting on burden of illness of IH, including:   - **Epidemiology**   - Incidence, prevalence, classification, subgroups, risk factors - **Presentation**   - Diagnostic criteria and patterns - **Clinical burden**    - Natural history, complications, comorbidities - **Treatment**   - Guidelines, landscape, real-world and trial outcomes, ongoing clinical trial landscape/emerging therapies, treatment patterns, adherence by clinicians to guidelines and by patients to therapy - **Humanistic burden**   - Health-related quality of life, patient preferences, treatment satisfaction, functional status and disability, caregiver burden, activities of daily living, utilities - **Economic burden**    - Productivity, absenteeism, presenteeism, direct and indirect costs, resource utilization, economic models and cost-effectiveness - **Data gaps and unmet needs** | Articles that are not about IH, or are partly on IH but do not report information on IH separately from other conditions |
| Publications with abstracts linked to a full-text manuscript; clinicaltrials.gov entries; guidelines; conference abstracts linked to a poster | Editorials, book reviews, letters, and other articles without abstracts, except for clinicaltrials.gov entries and guidelines; conference abstracts without posters |
| Primary studies, systematic reviews, meta-analyses, and guidelines | Case studies, case series, methods papers, narrative reviews, and other publications that are not primary studies, systematic reviews, meta-analyses, or guidelines |
| Primary studies, systematic reviews, meta-analyses, and guidelines from the last 10 years; conference abstracts from the last two years or meetings of that conference | Primary studies, systematic reviews, meta-analyses, and guidelines published prior to 10 years before the search (although older, seminal articles will be discussed for context); narrative reviews will also be excluded but discussed for context |
| Articles on humans | Animal or *in vitro* studies |
| Articles written in English or French | Articles written in languages other than English or French will be evaluated for translation |

**Table S4.** Included studies.

| **Study number** | **First author, year of publication** | **Publication type** | **IH diagnosis method** | **Study quality (Oxford levels)** |
| --- | --- | --- | --- | --- |
| 1 | Acquavella, 2020^1^ | Retrospective cohort study | NR | 2b |
| 2 | Anniss, 2016^2^ | Prospective cohort study | MSLT MSL ≤8 min and <2 SOREMPs | 2b |
| 3 | Barateau, 2016^3^ | Prospective case-control study | ICSD-3 | 2b |
| 4 | Barateau, 2017^4^ | Prospective case-control study | ICSD-3 | 2b |
| 5 | Bijlenga, 2021^5^ | Prospective cohort study | ICSD-3 | 2b |
| 6 | Bin-Hasan, 2018^6^ | Retrospective cross-sectional study | ICSD-3 | 2b |
| 7 | Bozluolçay, 2015^7^ | Retrospective cohort study | Latency of ≤8 in MSLT and 0 SOREMP | 2b |
| 8 | Busková, 2021^8^ | Prospective cross-sectional study | MSLT MSL ≤8 min and TST during 24-hours confirmed by PSG ≥660 minutes | 2b |
| 9 | Cairns, 2019^9^ | Retrospective cross-sectional study | MSLT MSL ≤8 min and ≤1 SOREMP | 2b |
| 10 | Chabani, 2020^10^ | Retrospective and prospective cohort study | Sleep time >11 h during 18–19 h long sleep monitoring or MSLT MSL <8 min and ≤1 SOREMP | 2b |
| 11 | Dauvilliers, 2012^11^ | Cross-sectional cohort study | ICSD-2 | 2b |
| 12 | Dauvilliers, 2019^12^ | Cross-sectional cohort study | MSLT MSL ≤8 minutes and ≤2 SOREMPs or TST ≥11 hours during the 24-hour PSG monitoring or TST ≥19 hours during a controlled 32-hour bed rest protocol | 2b |
| 13 | Dauvilliers, 2022^13^ | Randomized controlled trial | ICSD-2 or ICSD-3 | 1b |
| 14 | DelRosso, 2013^14^ | Retrospective cohort study | ICSD-2 | 2b |
| 15 | DelRosso, 2014^15^ | Retrospective cohort study | ICSD-2 | 2b |
| 16 | Deuschle, 2018^16^ | Prospective cohort study | ICSD-2 | 2b |
| 17 | Dietmann, 2021^17^ | Retrospective cohort study | ICSD-3, ICSD-2, or earlier versions of ICSD | 2b |
| 18 | Drakatos, 2013^18^ | Retrospective cohort study | ICSD-2 | 2b |
| 19 | Drakatos, 2013^19^ | Retrospective cohort study | ICSD-2 | 2b |
| 20 | Dzodzomenyo, 2015^20^ | Retrospective cohort study | MSLT MSL <8 min and <2 SOREMPs | 2b |
| 21 | Erdem, 2013^21^ | Retrospective cohort study | ICSD-2 | 2b |
| 22 | Evangelista, 2018^22^ | Prospective cohort study | ICSD-3 | 2b |
| 23 | Evangelista, 2021^23^ | Prospective cohort study | Objective hypersomnia and/or EDS, no medical  and psychiatric conditions and normal PSG parameters | 2b |
| 24 | Evangelista, 2022^24^ | Prospective cohort study | ICSD-3 current and alternative validated criteria, i.e. complaint of EDS and/or EQS, MSL ≤8 min and <2 SOREMPs on the standard MSLT and/or a TST ≥19 h on the 32-h bed-rest recording | 2b |
| 25 | Ferri, 2016^25^ | Prospective cohort study | ICSD-3 | 2b |
| 26 | Filardi, 2015^26^ | Prospective cohort study | ICSD-3 | 2b |
| 27 | Filardi, 2021^27^ | Systematic literature review of analytical cross-sectional studies | ICSD-3 for all 4 studies including [73, 95] | 2a |
| 28 | Holm, 2014^28^ | Prospective cohort study | ICSD-3 | 2b |
| 29 | Honda, 2022^29^ | Prospective cohort study | Criteria of EDS in ICSD-3 as pathological sleep prolongation (24-h PSG TST ≥ 660 min) without other sleep pathologies | 2b |
| 30 | Imanishi, 2020^30^ | Retrospective and prospective cohort study | ICSD-3 | 2b |
| 31 | Inoue, 2021^31^ | Randomized controlled trial | ICSD-2 | 1b |
| 32 | Ito, 2017^32^ | Prospective cohort study | ICSD-3 | 2b |
| 33 | Jagadish, 2021^33^ | Retrospective cohort study | ICSD-3 | 2b |
| 34 | Jaumally, 2021^34^ | Retrospective cohort study | ICSD-3; MSL <8min and <2 SOREMPs | 2b |
| 35 | Jennum, 2017^35^ | Prospective cohort study | ICSD-3 | 2b |
| 36 | Kim, 2016^36^ | Retrospective observational study | ICSD-2 | 2b |
| 37 | Kosky, 2016^37^ | Retrospective cohort study | ICSD-2 | 2b |
| 38 | Krahn, 2021^38^ | Guidelines | NA |  |
| 39 | Kretzschmar, 2016^39^ | Retrospective cohort study | ICSD-2 initially, then re-evaluated with ICSD-3 | 2b |
| 40 | Lee, 2017^40^ | Retrospective cohort study | ICSD-2 | 2b |
| 41 | Leu-Semenescu, 2014^41^ | Retrospective cohort study | (i) complaint of EDS occurring daily >3 mths and (ii) hypersomnia that was not better explained by another sleep disorder, medical or psychiatric condition, medication use or substance abuse, or behaviorally induced ISS. IH without long sleep time: MSLT MSL <8 min and ≤1 SOREMP. IH with long sleep time: TST >660 min (i.e. 11/24 h) during long-term sleep monitoring or night-time sleep time >600 min plus mean daytime sleep latencies <8 min for those who had no long-term sleep monitoring | 2b |
| 42 | Leu-Semenescu, 2016^42^ | Retrospective cohort study | ICSD-3 | 2b |
| 43 | Leu-Semenescu, 2022^43^ | Prospective cohort study | ICSD-2 | 2b |
| 44 | Lloyd, 2012^44^ | Retrospective cohort study | NR | 2b |
| 45 | Lopez, 2017^45^ | Retrospective cohort study | ICSD-3 | 2b |
| 46 | Lopez, 2017^46^ | Guidelines | NA |  |
| 47 | Lopez, 2020^47^ | Prospective cross-sectional study | ICSD-3 | 2b |
| 48 | Ludwig, 2018^48^ | Systematic literature review | 2 studies with IH patients: NR | 2a |
| 49 | Maness, 2019^49^ | Retrospective cohort study | ICSD-3 | 2b |
| 50 | Maski, 2020^50^ | Retrospective cross-sectional study | ICSD-3 | 2b |
| 51 | Maski, 2021^51^ | Guidelines | NA |  |
| 52 | Maski, 2021^52^ | Retrospective cross-sectional study | ICSD-3 | 2b |
| 53 | Maski, 2021^53^ | Systematic literature review and meta-analysis | 5 of the 10 IH studies: see [41, 42, 55, 87, 88] | 1a |
| 54 | Mathis, 2022^54^ | Retrospective cross-sectional study | ICSD-1 up to 2005, ICSD-2 up to 2014, and ICSD-3 since, with IH referring to subgroup with prolonged sleep need (>10 hours night sleep) | 2b |
| 55 | Mayer, 2015^55^ | Randomized controlled trial | ICSD-2 | 1b |
| 56 | Miglis, 2020^56^ | Prospective cohort study | ICSD-3 | 2b |
| 57 | Neikrug, 2017^57^ | Cross-sectional study | NR | 2b |
| 58 | Nevsimalova, 2021^58^ | Prospective cohort study | ICSD-2 or ICSD-3 | 2b |
| 59 | Nigam, 2022^59^ | Prospective cohort study | ICSD-3 | 2b |
| 60 | Nittur, 2013^60^ | Retrospective cohort study | ICSD-2 | 2b |
| 61 | Ong, 2020^61^ | Clinical controlled trial | Patient self-report | 1b |
| 62 | Ozaki, 2012^62^ | Prospective cohort study | ICSD-2 | 2b |
| 63 | Pascoe, 2019^63^ | Retrospective cohort study | ICSD-2 | 2b |
| 64 | Peter-Derex, 2013^64^ | Prospective cohort study | ICSD-2 | 2b |
| 65 | Peter-Derex, 2020^65^ | Retrospective cohort study | ICSD-3 | 2b |
| 66 | Philip, 2013^66^ | Prospective cohort study | ICSD-2 | 2b |
| 67 | Philip, 2014^67^ | Randomized controlled trial | ICSD-2 | 1b |
| 68 | Philip, 2021^68^ | Prospective case-control study | NR | 2b |
| 69 | Pizza, 2013^69^ | Prospective cohort study | ICSD-2 | 2b |
| 70 | Pizza, 2015^70^ | Cross-sectional study | ICSD-3 | 2b |
| 71 | Pizza, 2015^71^ | Cross-sectional study | ICSD-3 | 2b |
| 72 | Plante, 2018^72^ | Systematic literature review and meta-analysis | 1 of the 11 IH studies: see [66] | 2a |
| 73 | Ramm, 2019^73^ | Prospective cohort study | ICSD-3 | 2b |
| 74 | Rassu, 2022^74^ | Prospective and cross-sectional study | ICSD-3 | 2b |
| 75 | Ruoff, 2018^75^ | Retrospective cohort study | ICSD-3 | 2b |
| 76 | Sagaspe, 2019^76^ | Randomized controlled trial | ICSD-3 | 1b |
| 77 | Sasai-Sakuma, 2015^77^ | Prospective cohort study | ICSD-2 | 2b |
| 78 | Sasai-Sakuma, 2015^78^ | Retrospective cross-sectional study | ICSD-2 | 2b |
| 79 | Schinkelshoek, 2020^79^ | Prospective cohort study | ICSD-3 | 2b |
| 80 | Šonka, 2015^80^ | Retrospective cohort study | ICSD-2 | 2b |
| 81 | Sowa, 2016^81^ | Systematic literature review | NR | 2a |
| 82 | Suzuki, 2015^82^ | Prospective and cross-sectional study | ICSD-2 | 2b |
| 83 | Takei, 2012^83^ | Prospective cohort study | ICSD-2 | 2b |
| 84 | Thakrar, 2018^84^ | Retrospective cohort study | ICSD-3 | 2b |
| 85 | Trivedi, 2019^85^ | Prospective cohort study | ICSD-3 | 2b |
| 86 | Trotti, 2013^86^ | Retrospective cohort study | ICSD-2 | 2b |
| 87 | Trotti, 2015^87^ | Randomized controlled trial | ICSD-2 | 1b |
| 88 | Trotti, 2016^88^ | Retrospective cohort study | ICSD-3 | 2b |
| 89 | Trotti, 2017^89^ | Retrospective cohort study | ICSD-3 | 2b |
| 90 | Trotti, 2020^90^ | Cross-sectional study | Patient self-report | 2b |
| 91 | Trotti, 2021^91^ | Systematic literature review and meta-analysis | ICSD-2 for all 3 studies, see [31, 55, 87] | 1a |
| 92 | Trotti, 2022^92^ | Prospective cohort study | ICSD-3 | 2b |
| 93 | van der Sluiszen, 2021^93^ | Retrospective and prospective cohort study | ICSD-3 | 2b |
| 94 | van Holst, 2016^94^ | Prospective and cross-sectional study | ICSD-3 | 3b |
| 95 | Van Schie, 2012^95^ | Prospective cross-sectional study | ICSD-2 | 1b |
| 96 | Wang, 2021^96^ | Cross-sectional study | ICSD-2 initially, then regrouped with ICSD-3 | 2b |
| 97 | Wasling, 2020^97^ | Prospective and cross-sectional study | ICSD-3 | 1b |

**References**

1. Acquavella J, Mehra R, Bron M, Suomi JM, Hess GP. Prevalence of narcolepsy and other sleep disorders and frequency of diagnostic tests from 2013-2016 in insured patients actively seeking care. *J Clin Sleep Med.* 2020;16(8):1255-1263.

2. Anniss AM, Young A, O'Driscoll DM. Importance of urinary drug screening in the multiple seep latency test and maintenance of wakefulness test. *J Clin Sleep Med.* 2016;12(12):1633-1640.

3. Barateau L, Jaussent I, Lopez R, et al. Smoking, alcohol, drug use, abuse and dependence in narcolepsy and idiopathic hypersomnia: a case-control study. *Sleep.* 2016;39(3):573-580.

4. Barateau L, Lopez R, Arnulf I, et al. Comorbidity between central disorders of hypersomnolence and immune-based disorders. *Neurology.* 2017;88(1):93-100.

5. Bijlenga D, Urbanus B, van der Sluiszen N, et al. Comparing objective wakefulness and vigilance tests to on-the-road driving performance in narcolepsy and idiopathic hypersomnia. *J Sleep Res.* 2021:e13518.

6. Bin-Hasan S, Videnovic A, Maski K. Nocturnal REM sleep without atonia is a diagnostic biomarker of pediatric narcolepsy. *J Clin Sleep Med.* 2018;14(2):245-252.

7. Bozluolçay M, Nalbantp lu M, Benbir Senel G, Karadeniz D. What does one sleep-onset REM period - during either nocturnal polysomnography or multiple sleep latency test - mean in differential diagnosis of central hypersomnias? *Journal of Clinical Neurophysiology.* 2015;32:36T 368.

8. Busková J, Novák TDkB, Miletínová E, et al. Subjective symptoms and objective measures in idiopathic hypersomnia and hypersomnia associated with psychiatric disorders: a prospective cross-sectional study. *Journal of clinical sleep medicine.* 2021.

9. Cairns A, Bogan R. Comparison of the macro and microstructure of sleep in a sample of sleep clinic hypersomnia cases. *Neurobiol Sleep Circadian Rhythms.* 2019;6:62-69.

10. Chabani E, Vionnet MC, Beauté R, Leu-Semenescu S, Dodet P, Arnulf I. Blackout of my nights: Contentless, timeless and selfless report from the night in patients with central hypersomnias. *Consciousness and Cognition.* 2020;81.

11. Dauvilliers Y, Delallée N, Jaussent I, et al. Normal cerebrospinal fluid histamine and tele-methylhistamine levels in hypersomnia conditions. *Sleep.* 2012;35(10):1359-1366.

12. Dauvilliers Y, Evangelista E, Barateau L, et al. Measurement of symptoms in idiopathic hypersomnia: The Idiopathic Hypersomnia Severity Scale. *Neurology.* 2019;92(15):e1754-e1762.

13. Dauvilliers Y, Arnulf I, Foldvary-Schaefer N, et al. Safety and efficacy of lower-sodium oxybate in adults with idiopathic hypersomnia: a phase 3, placebo-controlled, double-blind, randomised withdrawal study. *Lancet Neurol.* 2022;21(1):53-65.

14. DelRosso LM, Chesson AL, Jr., Hoque R. Characterization of REM sleep without atonia in patients with narcolepsy and idiopathic hypersomnia using AASM scoring manual criteria. *J Clin Sleep Med.* 2013;9(7):675-680.

15. DelRosso LM, Chesson AL, Hoque R. Manual characterization of sleep spindle index in patients with narcolepsy and idiopathic hypersomnia. *Sleep Disord.* 2014;2014:271802.

16. Deuschle M, Schredl M, Wisch C, et al. Serum brain-derived neurotrophic factor (BDNF) in sleep-disordered patients: relation to sleep stage N3 and rapid eye movement (REM) sleep across diagnostic entities. *J Sleep Res.* 2018;27(1):73-77.

17. Dietmann A, Gallino C, Wenz E, Mathis J, Bassetti CLA. Multiple sleep latency test and polysomnography in patients with central disorders of hypersomnolence. *Sleep Med.* 2021;79:6-10.

18. Drakatos P, Kosky CA, Higgins SE, Muza RT, Williams AJ, Leschziner GD. First rapid eye movement sleep periods and sleep-onset rapid eye movement periods in sleep-stage sequencing of hypersomnias. *Sleep Med.* 2013;14(9):897-901.

19. Drakatos P, Suri A, Higgins SE, et al. Sleep stage sequence analysis of sleep onset REM periods in the hypersomnias. *J Neurol Neurosurg Psychiatry.* 2013;84(2):223-227.

20. Dzodzomenyo S, Stolfi A, Splaingard D, Earley E, Onadeko O, Splaingard M. Urine toxicology screen in multiple sleep latency test: the correlation of positive tetrahydrocannabinol, drug negative patients, and narcolepsy. *J Clin Sleep Med.* 2015;11(2):93-99.

21. Erdem M, Bolu A, Ünlü AG, Alper M, Yetkin S. Comparison of polysomnography and multiple sleep latency test findings in subjects with narcolepsy and İdiopathic hypersomnia. *Noro Psikiyatr Ars.* 2013;50(3):252-255.

22. Evangelista E, Lopez R, Barateau L, et al. Alternative diagnostic criteria for idiopathic hypersomnia: A 32-hour protocol. *Ann Neurol.* 2018;83(2):235-247.

23. Evangelista E, Rassu AL, Barateau L, et al. Characteristics associated with hypersomnia and excessive daytime sleepiness identified by extended polysomnography recording. *Sleep.* 2021;44(5).

24. Evangelista E, Rassu AL, Lopez R, et al. Sleep inertia measurement with the psychomotor vigilance task in idiopathic hypersomnia. *Sleep.* 2022:1-12.

25. Ferri R, Pizza F, Vandi S, Iloti M, Plazzi G. Decreased sleep stage transition pattern complexity in narcolepsy type 1. *Clin Neurophysiol.* 2016;127(8):2812-2819.

26. Filardi M, Pizza F, Martoni M, Vandi S, Plazzi G, Natale V. Actigraphic assessment of sleep/wake behavior in central disorders of hypersomnolence. *Sleep Med.* 2015;16(1):126-130.

27. Filardi M, D'Anselmo A, Agnoli S, et al. Cognitive dysfunction in central disorders of hypersomnolence: A systematic review. *Sleep medicine reviews.* 2021;59:101510.

28. Holm A, Bang-Berthelsen CH, Knudsen S, et al. MiRNA profiles in cerebrospinal fluid from patients with central hypersomnias. *J Neurol Sci.* 2014;347(1-2):199-204.

29. Honda M, Kimura S, Sasaki K, Wada M, Ito W. Absence of multiple sleep-onset rapid eye movement periods (SOREMPs) is not a specific feature of patients with pathological sleep prolongation. *Sleep and Biological Rhythms.* 2022;20:107-114.

30. Imanishi A, Kawazoe T, Hamada Y, et al. Early detection of Niemann-pick disease type C with cataplexy and orexin levels: continuous observation with and without Miglustat. *Orphanet J Rare Dis.* 2020;15(1):269.

31. Inoue Y, Tabata T, Tsukimori N. Efficacy and safety of modafinil in patients with idiopathic hypersomnia without long sleep time: a multicenter, randomized, double-blind, placebo-controlled, parallel-group comparison study. *Sleep Med.* 2021;80:315-321.

32. Ito W, Honda M, Ueno T, Kato N. Hypersomnia with ADHD: a possible subtype of narcolepsy type 2. *Sleep and Biological Rhythms.* 2017;16:205-210.

33. Jagadish S, Singer W, Kotagal S. Autonomic dysfunction in childhood hypersomnia disorders. *Sleep Med.* 2021;78:43-48.

34. Jaumally BA, Das A, Cassell NC, et al. Excessive daytime sleepiness in cancer patients. *Sleep Breath.* 2021;25(2):1063-1067.

35. Jennum PJ, Østergaard Pedersen L, Czarna Bahl JM, et al. Cerebrospinal fluid biomarkers of neurodegeneration are decreased or normal in narcolepsy. *Sleep.* 2017;40(1).

36. Kim T, Lee JH, Lee CS, Yoon IY. Different fates of excessive daytime sleepiness: survival analysis for remission. *Acta Neurol Scand.* 2016;134(1):35-41.

37. Kosky CA, Bonakis A, Yogendran A, Hettiarachchi G, Dargan PI, Williams AJ. Urine toxicology in adults evaluated for a central hypersomnia and how the results modify the physician's diagnosis. *J Clin Sleep Med.* 2016;12(11):1499-1505.

38. Krahn LE, Arand DL, Avidan AY, et al. Recommended protocols for the multiple sleep latency test and maintenance of wakefulness test in adults: guidance from the American Academy of Sleep Medicine. *J Clin Sleep Med.* 2021;17(12):2489-2498.

39. Kretzschmar U, Werth E, Sturzenegger C, Khatami R, Bassetti CL, Baumann CR. Which diagnostic findings in disorders with excessive daytime sleepiness are really helpful? A retrospective study. *J Sleep Res.* 2016;25(3):307-313.

40. Lee J, Na G, Joo EY, Lee M, Lee J. Clinical and polysomnographic characteristics of excessive daytime sleepiness in children. *Sleep Breath.* 2017;21(4):967-974.

41. Leu-Semenescu S, Nittur N, Golmard JL, Arnulf I. Effects of pitolisant, a histamine H3 inverse agonist, in drug-resistant idiopathic and symptomatic hypersomnia: a chart review. *Sleep Med.* 2014;15(6):681-687.

42. Leu-Semenescu S, Louis P, Arnulf I. Benefits and risk of sodium oxybate in idiopathic hypersomnia versus narcolepsy type 1: a chart review. *Sleep Med.* 2016;17:38-44.

43. Leu-Semenescu S, Maranci J-B, Lopez R, et al. Comorbid parasomnias in narcolepsy and idiopathic hypersomnia: more REM than NREM parasomnias. *Journal of clinical sleep medicine.* 2022.

44. Lloyd R, Tippmann-Peikert M, Slocumb N, Kotagal S. Characteristics of REM sleep behavior disorder in childhood. *J Clin Sleep Med.* 2012;8(2):127-131.

45. Lopez R, Doukkali A, Barateau L, et al. Test-retest reliability of the multiple sleep latency test in central disorders of hypersomnolence. *Sleep.* 2017;40(12).

46. Lopez R, Arnulf I, Drouot X, Lecendreux M, Dauvilliers Y. French consensus. Management of patients with hypersomnia: Which strategy? *Rev Neurol (Paris).* 2017;173(1-2):8-18.

47. Lopez R, Micoulaud-Franchi JA, Camodeca L, Gachet M, Jaussent I, Dauvilliers Y. Association of inattention, hyperactivity, and hypersomnolence in two clinic-based adult cohorts. *J Atten Disord.* 2020;24(4):555-564.

48. Ludwig B, Smith S, Heussler H. Associations between neuropsychological, neurobehavioral and emotional functioning and either narcolepsy or idiopathic hypersomnia in children and adolescents. *J Clin Sleep Med.* 2018;14(4):661-674.

49. Maness C, Saini P, Bliwise DL, Olvera V, Rye DB, Trotti LM. Systemic exertion intolerance disease/chronic fatigue syndrome is common in sleep centre patients with hypersomnolence: A retrospective pilot study. *J Sleep Res.* 2019;28(3):e12689.

50. Maski K, Pizza F, Liu S, et al. Defining disrupted nighttime sleep and assessing its diagnostic utility for pediatric narcolepsy type 1. *Sleep.* 2020;43(10).

51. Maski K, Trotti LM, Kotagal S, et al. Treatment of central disorders of hypersomnolence: an American Academy of Sleep Medicine clinical practice guideline. *J Clin Sleep Med.* 2021;17(9):1881-1893.

52. Maski KP, Colclasure A, Little E, et al. Stability of nocturnal wake and sleep stages defines central nervous system disorders of hypersomnolence. *Sleep.* 2021;44(7).

53. Maski K, Trotti LM, Kotagal S, et al. Treatment of central disorders of hypersomnolence: an American Academy of Sleep Medicine systematic review, meta-analysis, and GRADE assessment. *J Clin Sleep Med.* 2021;17(9):1895-1945.

54. Mathis J, Andres D, Schmitt WJ, Bassetti CLA, Hess CW, Schreier DR. The diagnostic value of sleep and vigilance tests in central disorders of hypersomnolence. *Sleep.* 2022.

55. Mayer G, Benes H, Young P, Bitterlich M, Rodenbeck A. Modafinil in the treatment of idiopathic hypersomnia without long sleep time--a randomized, double-blind, placebo-controlled study. *J Sleep Res.* 2015;24(1):74-81.

56. Miglis MG, Schneider L, Kim P, Cheung J, Trotti LM. Frequency and severity of autonomic symptoms in idiopathic hypersomnia. *J Clin Sleep Med.* 2020;16(5):749-756.

57. Neikrug AB, Crawford MR, Ong JC. Behavioral sleep medicine services for hypersomnia disorders: A survey study. *Behav Sleep Med.* 2017;15(2):158-171.

58. Nevsimalova S, Susta M, Prihodova I, Horvat EM, Milata M, Sonka K. Idiopathic hypersomnia: a homogeneous or heterogeneous disease? *Sleep Med.* 2021;80:86-91.

59. Nigam M, Hippolyte A, Dodet P, et al. Sleeping through a pandemic: impact of COVID-19-related restrictions on narcolepsy and idiopathic hypersomnia. *J Clin Sleep Med.* 2022;18(1):255-263.

60. Nittur N, Konofal E, Dauvilliers Y, et al. Mazindol in narcolepsy and idiopathic and symptomatic hypersomnia refractory to stimulants: a long-term chart review. *Sleep Med.* 2013;14(1):30-36.

61. Ong JC, Dawson SC, Mundt JM, Moore C. Developing a cognitive behavioral therapy for hypersomnia using telehealth: a feasibility study. *J Clin Sleep Med.* 2020;16(12):2047-2062.

62. Ozaki A, Inoue Y, Hayashida K, et al. Quality of life in patients with narcolepsy with cataplexy, narcolepsy without cataplexy, and idiopathic hypersomnia without long sleep time: comparison between patients on psychostimulants, drug-naïve patients and the general Japanese population. *Sleep Med.* 2012;13(2):200-206.

63. Pascoe M, Bena J, Foldvary-Schaefer N. Effects of pharmacotherapy treatment on patient-reported outcomes in a narcolepsy and idiopathic hypersomnia cohort. *J Clin Sleep Med.* 2019;15(12):1799-1806.

64. Peter-Derex L, Perrin F, Petitjean T, Garcia-Larrea L, Bastuji H. Discriminating neurological from psychiatric hypersomnia using the forced awakening test. *Neurophysiologie Clinique/Clinical Neurophysiology.* 2013;43:171-179.

65. Peter-Derex L, Subtil F, Lemaître G, et al. Observation and interview-based diurnal sleepiness inventory for measurement of sleepiness in patients referred for narcolepsy or idiopathic hypersomnia. *Journal of clinical sleep medicine : JCSM : official publication of the American Academy of Sleep Medicine.* 2020.

66. Philip P, Chaufton C, Taillard J, et al. Maintenance of Wakefulness Test scores and driving performance in sleep disorder patients and controls. *International journal of psychophysiology : official journal of the International Organization of Psychophysiology.* 2013;89 2:195-202.

67. Philip P, Chaufton C, Taillard J, et al. Modafinil improves real driving performance in patients with hypersomnia: a randomized double-blind placebo-controlled crossover clinical trial. *Sleep.* 2014;37 3:483-487.

68. Philip P, Guichard K, Strauss M, et al. Maintenance of wakefulness test: how does it predict accident risk in patients with sleep disorders? *Sleep medicine.* 2021(77).

69. Pizza F, Moghadam KK, Vandi S, et al. Daytime continuous polysomnography predicts MSLT results in hypersomnias of central origin. *J Sleep Res.* 2013;22(1):32-40.

70. Pizza F, Jaussent I, Lopez R, et al. Car crashes and central disorders of hypersomnolence: A French study. *PLoS One.* 2015;10(6):e0129386.

71. Pizza F, Vandi S, Iloti M, et al. Nocturnal Sleep Dynamics Identify Narcolepsy Type 1. *Sleep.* 2015;38(8):1277-1284.

72. Plante DT. Nocturnal sleep architecture in idiopathic hypersomnia: a systematic review and meta-analysis. *Sleep Med.* 2018;45:17-24.

73. Ramm M, Boentert M, Lojewsky N, Jafarpour A, Young P, Heidbreder A. Disease-specific attention impairment in disorders of chronic excessive daytime sleepiness. *Sleep Med.* 2019;53:133-140.

74. Rassu AL, Evangelista E, Barateau L, et al. Idiopathic Hypersomnia Severity Scale to better quantify symptoms severity and their consequences in idiopathic hypersomnia. *Journal of clinical sleep medicine : JCSM : official publication of the American Academy of Sleep Medicine.* 2022.

75. Ruoff C, Pizza F, Trotti LM, et al. The MSLT is repeatable in narcolepsy Type 1 but not narcolepsy type 2: A retrospective patient study. *J Clin Sleep Med.* 2018;14(1):65-74.

76. Sagaspe P, Micoulaud-Franchi JA, Coste O, et al. Maintenance of Wakefulness Test, real and simulated driving in patients with narcolepsy/hypersomnia. *Sleep Med.* 2019;55:1-5.

77. Sasai-Sakuma T, Inoue Y. Differences in electroencephalographic findings among categories of narcolepsy-spectrum disorders. *Sleep Med.* 2015;16(8):999-1005.

78. Sasai-Sakuma T, Kinoshita A, Inoue Y. Polysomnographic Assessment of Sleep Comorbidities in Drug-Naïve Narcolepsy-Spectrum Disorders--A Japanese Cross-Sectional Study. *PLoS One.* 2015;10(8):e0136988.

79. Schinkelshoek MS, de Wit K, Bruggink V, Fronczek R, Lammers GJ. Daytime sleep state misperception in a tertiary sleep centre population. *Sleep Med.* 2020;69:78-84.

80. Šonka K, Šusta M, Billiard M. Narcolepsy with and without cataplexy, idiopathic hypersomnia with and without long sleep time: a cluster analysis. *Sleep Med.* 2015;16(2):225-231.

81. Sowa NA. Idiopathic hypersomnia and hypersomnolence disorder: A systematic review of the literature. *Psychosomatics.* 2016;57(2):152-164.

82. Suzuki K, Miyamoto M, Miyamoto T, et al. The prevalence and characteristics of primary headache and dream-enacting behaviour in japanese patients with narcolepsy or idiopathic hypersomnia: A multi-centre cross-sectional study. *PLoS One.* 2015;10(9):e0139229.

83. Takei Y, Komada Y, Namba K, et al. Differences in findings of nocturnal polysomnography and multiple sleep latency test between narcolepsy and idiopathic hypersomnia. *Clin Neurophysiol.* 2012;123(1):137-141.

84. Thakrar C, Patel K, D'Ancona G, et al. Effectiveness and side-effect profile of stimulant therapy as monotherapy and in combination in the central hypersomnias in clinical practice. *J Sleep Res.* 2018;27(4):e12627.

85. Trivedi M, Sreedharan SE, Nair SN, et al. Central disorders of hypersomnolence in children and adults: A comparative study from South India. *Ann Indian Acad Neurol.* 2019;22(4):442-446.

86. Trotti LM, Staab BA, Rye DB. Test-retest reliability of the multiple sleep latency test in narcolepsy without cataplexy and idiopathic hypersomnia. *J Clin Sleep Med.* 2013;9(8):789-795.

87. Trotti LM, Saini P, Bliwise DL, Freeman AA, Jenkins A, Rye DB. Clarithromycin in γ-aminobutyric acid-Related hypersomnolence: A randomized, crossover trial. *Ann Neurol.* 2015;78(3):454-465.

88. Trotti LM, Saini P, Koola C, LaBarbera V, Bliwise DL, Rye DB. Flumazenil for the treatment of refractory hypersomnolence: clinical experience with 153 patients. *J Clin Sleep Med.* 2016;12(10):1389-1394.

89. Trotti LM, Bliwise DL. Brain MRI findings in patients with idiopathic hypersomnia. *Clin Neurol Neurosurg.* 2017;157:19-21.

90. Trotti LM, Ong JC, Plante DT, Friederich Murray C, King R, Bliwise DL. Disease symptomatology and response to treatment in people with idiopathic hypersomnia: initial data from the Hypersomnia Foundation registry. *Sleep Med.* 2020;75:343-349.

91. Trotti LM, Becker LA, Friederich Murray C, Hoque R. Medications for daytime sleepiness in individuals with idiopathic hypersomnia. *The Cochrane database of systematic reviews.* 2021;5:CD012714.

92. Trotti LM, Saini P, Bremer E, et al. The Psychomotor Vigilance Test as a measure of alertness and sleep inertia in people with central disorders of hypersomnolence. *J Clin Sleep Med.* 2022.

93. van der Sluiszen N, Urbanus B, Lammers GJ, Overeem S, Ramaekers JG, Vermeeren A. On-the-road driving performance of patients with central disorders of hypersomnolence. *Traffic Inj Prev.* 2021;22(2):120-126.

94. van Holst RJ, van der Cruijsen L, van Mierlo P, et al. Aberrant food choices after satiation in human orexin-deficient narcolepsy type 1. *Sleep.* 2016;39(11):1951-1959.

95. Van Schie MK, Thijs RD, Fronczek R, Middelkoop HA, Lammers GJ, Van Dijk JG. Sustained attention to response task (SART) shows impaired vigilance in a spectrum of disorders of excessive daytime sleepiness. *J Sleep Res.* 2012;21(4):390-395.

96. Wang P, Li Q, Dong X-s, et al. Lipocalin-type prostaglandin D synthase levels increase in patients with narcolepsy and idiopathic hypersomnia. *Sleep.* 2021:1-6.

97. Wasling HB, Bornstein A, Wasling P. Quality of life and procrastination in post-H1N1 narcolepsy, sporadic narcolepsy and idiopathic hypersomnia, a Swedish cross-sectional study. *Sleep Med.* 2020;76:104-112.
